# Variational Quantum Algorithm for Non-Markovian Quantum Dynamics Using an Ensemble of Ehrenfest Trajectories

**DOI:** 10.1021/acs.jpclett.4c03431

**Published:** 2025-01-22

**Authors:** Peter L. Walters, Mohammad U. Sherazi, Fei Wang

**Affiliations:** † Department of Chemistry and Biochemistry, George Mason University, Fairfax, Virginia 22030, United States; ‡ Department of Physics and Astronomy, George Mason University, Fairfax, Virginia 22030, United States; § Quantum Science and Engineering Center, George Mason University, Fairfax, Virginia 22030, United States

## Abstract

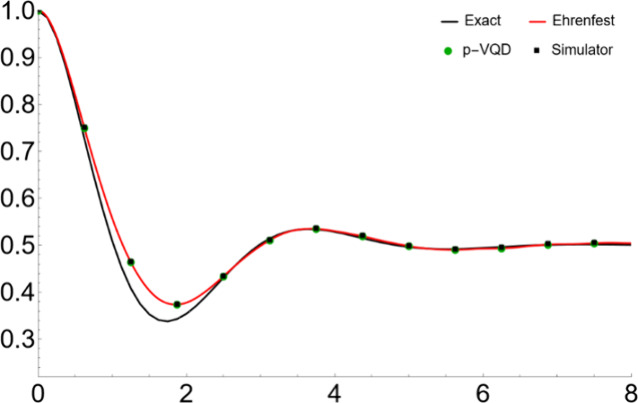

The simulation of non-Markovian quantum dynamics plays an important role in the understanding of charge and exciton dynamics in the condensed phase environment, yet such a simulation remains computationally expensive on classical computers. In this work, we develop a variational quantum algorithm that is capable of simulating non-Markovian quantum dynamics on quantum computers. The algorithm captures the non-Markovian effect by employing the Ehrenfest trajectories and Monte Carlo sampling of their thermal distribution. We test the algorithm with the spin-boson model on the quantum simulator, and the results match quantitatively with the exact ones. The algorithm naturally fits into the parallel computing platform of the NISQ devices and can be extended to anharmonic system-bath interactions and multistate systems.

The simulation of quantum dynamics in the condensed phase environment can offer critical insight into the charge and exciton transfer processes in solutions, functional materials, and biomolecules.^1−7^ A particularly challenging task is the simulation in the non-Markovian regime where the system’s past trajectory influences its present state. In such a case, the computation on the classical computers often scales exponentially with respect to the system size and the memory length. A quantum computer, on the other hand, can encode the exponential number of states with a linear number of qubits. Very recently, there has been a growing interest in the development of quantum algorithms for non-Markovian quantum dynamics.^8−15^ Given the current stage of the quantum devices, it is unlikely that the full-scale quantum algorithms such as Shor’s factoring^16^ can be implemented in the near future. Therefore, a hybrid quantum-classical approach that utilizes variational quantum circuits^17,18^ seems to be the practical and immediate application of near-term quantum computing. In this work, we present a variational quantum algorithm (VQA) for simulating non-Markovian quantum dynamics. The algorithm uses the ensemble-averaged Ehrenfest trajectories (EAET) to capture the non-Markovian effect and employs the “projected–Variational Quantum Dynamics” (p-VQD)^19^ method to parametrize the circuit. In what follows, we use a quantum system linearly coupled to its harmonic bath as our model.

The Hamiltonian of such can be written as
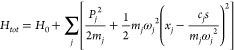
1where *H*_0_ is the system Hamiltonian, *s* and *x*_*j*_ denote the system and bath coordinates, respectively, and *c*_*j*_ denotes the system-bath coupling. The strength weighted density of modes defines the spectral density^20^
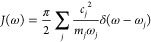
2

The system is perturbed by a time-dependent driving force from the bath^21^
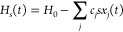
3where
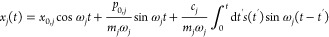
4

The nonlocal memory kernel in the last part of eq 4, termed the back-reaction^22^ (i.e., kicking back by the system), is partially responsible for the non-Markovian effect. The other contribution is from the integration of the phase space variables *x*_0, *j*_ and *p*_0, *j*_ of the bath. The exponential scaling due to the back-reaction can be easily seen with discretized position and time. For instance, for a two-state system, the trajectory proliferation can be pictorially represented as in Figure 1. Each trajectory of *s*(*t*′) gives a specific realization of *x*_*j*_(*t*).

**Figure 1:**
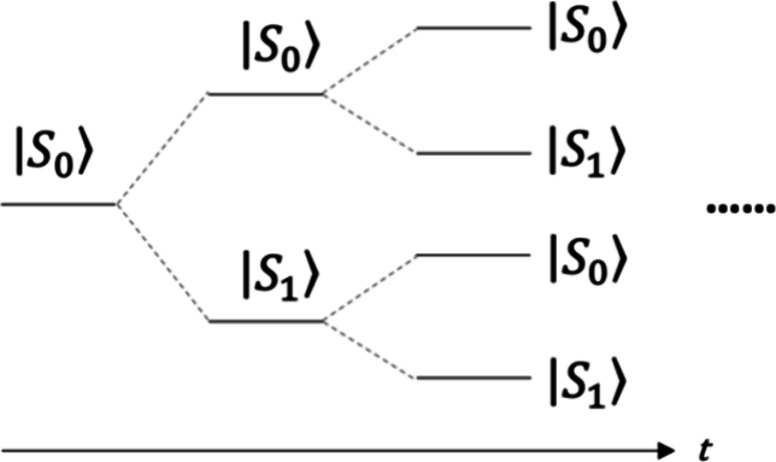
Exponential proliferation of trajectories due to the back-reaction.

In the path integral formulation, every trajectory contributes to the dynamics but with a different weight. In the Ehrenfest trajectory (ET) approximation, we replace all possible trajectories with one average trajectory. Specifically,
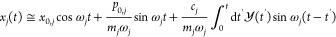
5where  is the average of all possible positions at time *t*′. For example, for a two-state system in which the two localized states are also position eigenstates (so-called DVR states^23^), , where *P*_0_(*t*′) and *P*_1_(*t*′) are the populations of these two states at time *t*′, and *s*_0_ and *s*_1_ are the position values. Within the ET approximation, the population dynamics can be solved by Markovian propagation
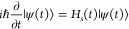
6and the population value of *P*(*t*′) is updated in eq 5 as time progresses. To incorporate the thermal effect, Monte Carlo sampling of **x**_0_ and **p**_0_ from the Wigner distribution, eq 7, is performed.
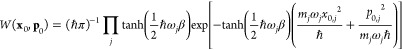
7

The result is the ensemble averaged |ψ(*t*)⟩, which we call the ensemble averaged Ehrenfest trajectory (EAET) approach. It draws inspirations from the ensemble averaged classical path (EACP) developed by Makri.^24^ One appealing aspect of these approaches is that they are not limited to the harmonic bath linearly coupled to the system; the framework can be equally adapted to nonlinear coupling and an anharmonic environment. The reason is that, for an anharmonic trajectory, the equation of motion can be written in a analogous way as in eq 4, which is composed of a free propagation part and a back-reaction part. The free propagation can be easily solved numerically for any potential. The back-reaction part arises from the system-bath interaction, and the analytical form is generally not known. However, we can well approximate the anharmonic back-reaction with the harmonic back-reaction (HBR) expressed in eq 4(22,25) due to the fact that the back-reaction is largely a zero-point energy effect associated with the bottom of the potential well,^26^ which in most cases can be well approximated to be harmonic. Therefore, the EAET approximation for the anharmonic bath is no different from the expression of eq 5, except to replace the free propagation part with the numerical anharmonic one. The anharmonic bath Wigner distribution can be obtained by methods such as adiabatic switching.^27^ We emphasize that the Wigner distribution automatically accounts for the zero-point energy effect of the bath, whereas the Boltzmann distribution does not.

To assess the validity of the EAET approximation, we make connections to the path integral formulation. By inserting eq 4 in the action integral and performing the integration over **x**_0_ and **p**_0_ with the Wigner distribution, we reproduce the Feynman-Vernon’s influence functional IF,^28^ in which
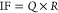
8

The influence functional alters the system’s free dynamics in the harmonic dissipative environment in an exact way. Here,
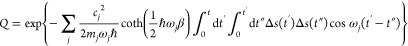
9and
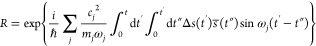
10where *s*^+^ and *s*^–^ denote the forward and backward position, respectively, with Δ*s* = *s*^+^–*s*^–^ and . Apparent from eqs 9 and 10 is the non-Markovian effect induced by the double time integral. The non-Markovianity in *Q* results from the integration of the phase space variables, whereas the non-Markovianity in *R* originates from the back-reaction term in eq 4. As pointed out by Makri, the temperature-dependent *Q* is related to the simulated emission and absorption of phonons, whereas *R*, the spontaneous emission.^26^ In the EAET approximation, *Q* remains unchanged and *R* becomes
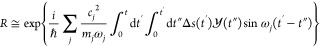
11

Therefore, the EAET approximation preserves most of the non-Markovian effect by the double-time integral as well as the quantum mechanical effect from the Wigner distribution and the back-reaction. The EAET approximation becomes more accurate as the temperature increases or the coupling strength becomes smaller, since in those regimes, the free propagation term in eq 5 dominates.

To propagate eq 6 on a quantum computer, we use the VQA approach. We adopt the p-VQD,^19^ an optimization-based method, to construct the quantum circuit. First, define the loss function *L* with the parametrized circuit *C*(**θ**) as
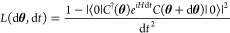
12in which the factor d*t*^2^ in the denominator is to ensure that *L* is independent of the time step size. If the parametrized circuit is composed of generalized Pauli operators, then the gradient can be computed exactly using the parameter shift rule^29,30^
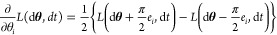
13where *e*_*i*_ is the vector in the *i*-th direction. In this work, we choose to use the ADAM stochastic algorithm for the gradient descent optimization.^31^ The advantages of the p-VQD compared to the time-dependent variational algorithm^32^ are that it circumvents the numerical instability arising from matrix inversion, it scales linearly with the number of parameters, and it avoids the barren plateaus.^19^

In the following, we use the spin-boson model to test the algorithm. The Hamiltonian in the EAET limit can be written as
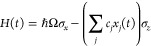
14in which *x*_*j*_(*t*) is given by eq 5. We choose the bath to have the Ohmic spectral density
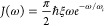
15where the dimensionless ξ is the Kondo parameter that determines the strength of the system-bath coupling and ω_*c*_ is the cutoff frequency. We use 60 oscillators of different frequencies in the numerical calculation, following the discretization procedure given by Walters et al.^33^ For a two-level system which requires one qubit, there exits an exact ansatz for the unitary operation that employs the *ZXZ* decomposition^34^
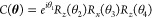
16

We show the simulation results in Figures 2–5. Specifically, Figures 2 and 3 show the population dynamics with parameters Ω = 1, ξ = 1.2, ω_*c*_ = 2.5, and β = 0.2. The system is initially in the reactant state. In Figure 2, the “Exact” result is from the numerically exact QuAPI calculation,^35^ the “Ehrenfest” result is obtained by numerically solving the differential eq 6 with the RK4 method and then averaging over the Monte Carlo points (i.e., EAET), the “p-VQD” result is obtained from numerically solving eq 6 with the parametrized circuit in eq 16 and the ADAM optimization, and the “Simulator” result is obtained by using the p-VQD approach and the measurement of the circuit with 50,000 shots. In Figure 3a–d, the “Ehrenfest 1 IC” is the simulation result of the ET approximation with one initial condition, **x**_0_ and **p**_0_, randomly chosen. The “Ehrenfest 10,000 ICs” is from the EAET, averaging over 10,000 initial conditions. Similarly, Figures 4 and 5 use parameters Ω = 1, ξ = 0.3, ω_*c*_ = 5, and β = 5. To facilitate better reading of the plots, we summarize the above-mentioned methods and results in Table 1. The comparison between “Exact” and “Ehrenfest” serves the purpose to gauge the accuracy of the EAET approximation; the comparison between “Ehrenfest” and “p-VQD” aims to assess the accuracy of the circuit ansatz and the gradient descent algorithm, and the comparison between “p-VQD” and “Simulator” gives the impression about the measurement shot noise.

**Table 1:** Results and Methods in the Figures

Result	Method
Exact	QuAPI^35^
Ehrenfest	EAET: numerically solving the differential eq 6 with the RK4 method and then averaging over the Monte Carlo points
p-VQD	Numerically solving eq 6 with the parametrized circuit in eq 16 and the ADAM optimization
Simulator	p-VQD approach and the circuit measurement with 50,000 shots
Ehrenfest 1 IC	ET approximation with one initial condition, **x**_0_ and **p**_0_, randomly chosen

**Figure 2:**
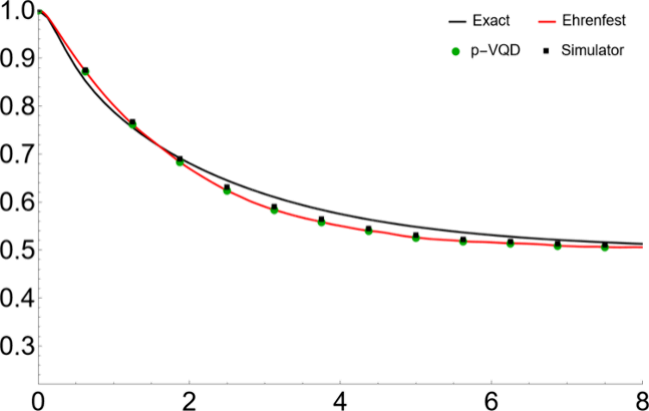
Population dynamics for a symmetric two-level system coupled to a harmonic bath with parameters Ω = 1, ξ = 1.2, ω_*c*_ = 2.5, and β = 0.2 and the system initially populated in the reactant state.

**Figure 3:**
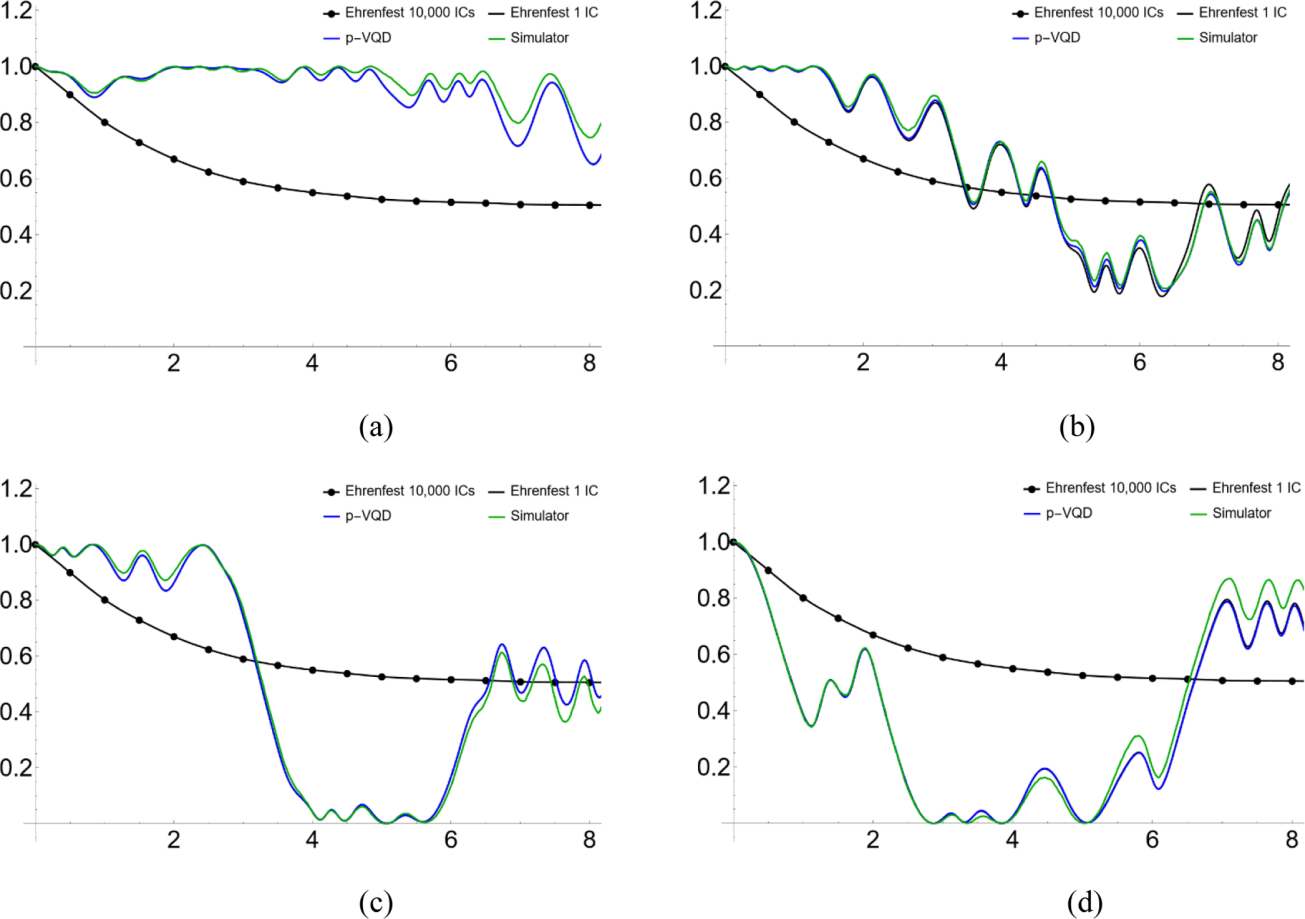
(a–d) Population dynamics for a symmetric two-level system coupled to a harmonic bath with one initial condition and parameters Ω = 1, ξ = 1.2, and ω_*c*_ = 2.5. The system is initially in the reactant state. (The “Ehrenfest 10,000 ICs” is for comparison purposes.)

**Figure 4:**
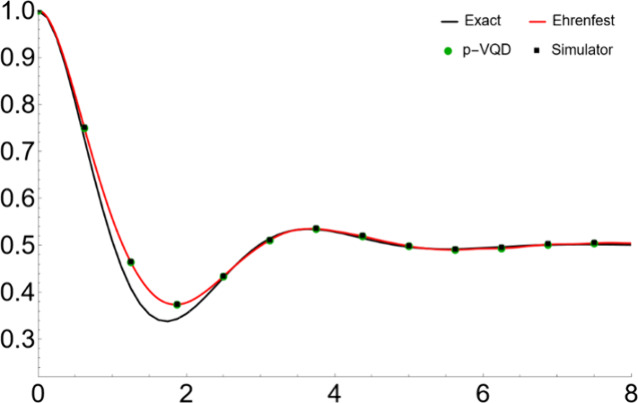
Population dynamics for a symmetric two-level system coupled to a harmonic bath with parameters Ω = 1, ξ = 0.3, ω_*c*_ = 5, and β = 5 and the system initially populated in the reactant state.

**Figure 5:**
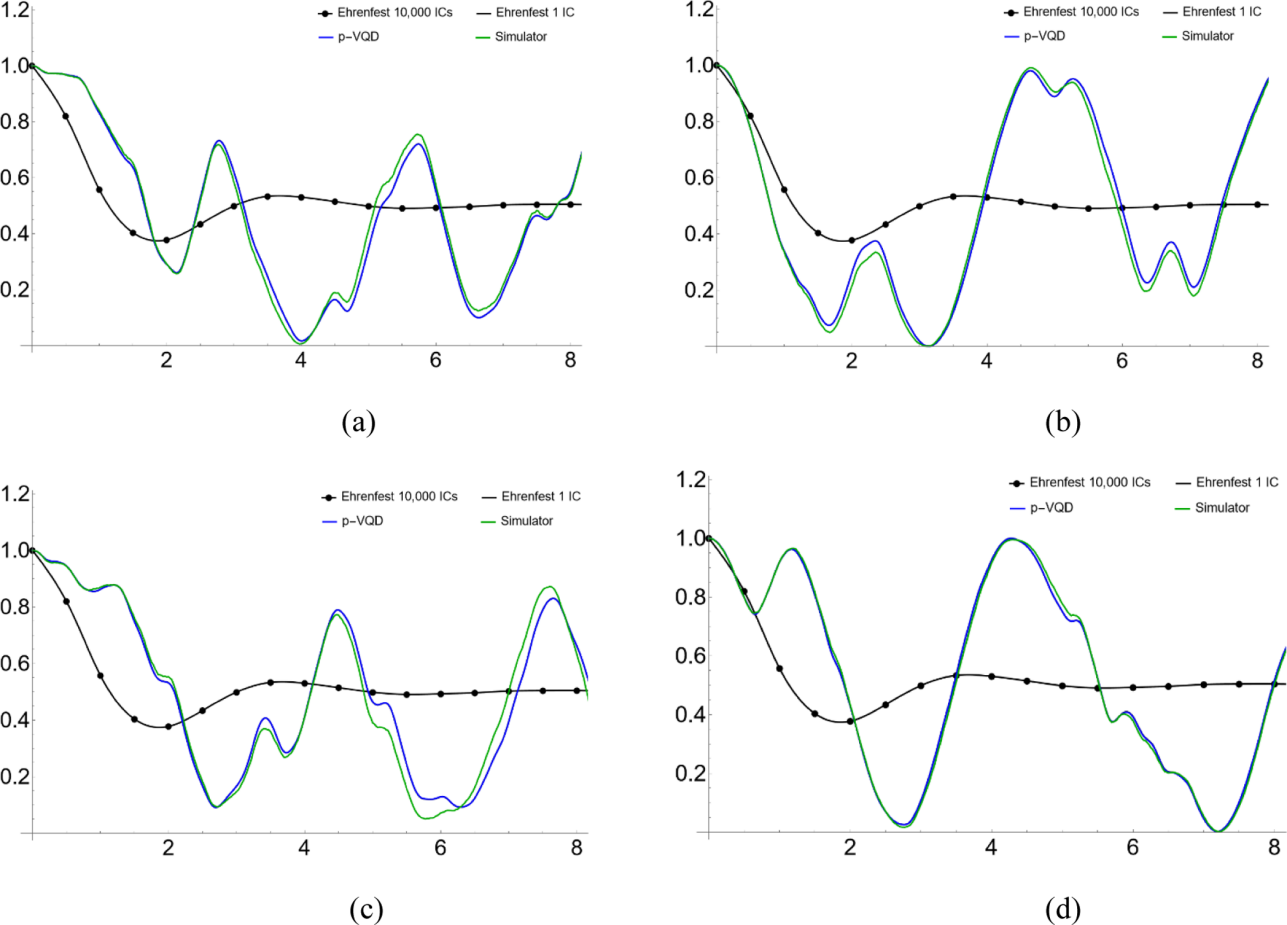
(a–d) Population dynamics for a symmetric two-level system coupled to a harmonic bath with one initial condition and parameters Ω = 1, ξ = 0.3, and ω_*c*_ = 5. The system is initially in the reactant state. (The “Ehrenfest 10,000 ICs” is for comparison purposes.)

In Figures 2 and 4, the “Ehrenfest” (i.e., EAET) matches well with the “Exact” benchmark. It captures the correct time scale and the dynamical behavior. The small discrepancy comes from the fact that the ET ignores some quantum interference effect from the different possible trajectories and therefore tends to decay more. However, due to the decoherence effect of the bath, the dynamics that follows the Eherenfest trajectory eventually becomes more classical, and the EAET results are still in quantitative agreement with the exact ones. Shown in Figures 3 and 5, for one initial condition, the “Simulator” result eventually deviates from the “p-VQD” result due to the shot noise. However, as demonstrated in Figure 2 and 4, the “Simulator” result matches well with the “p-VQD” result, by averaging over enough initial conditions. Therefore, by the ensemble averaging, the shot error can be reduced due to its random nature. Figures 3 and 5 also show that mild Monte Carlo points such as 10,000 will make the results converge well.

In summary, we have developed a variational quantum algorithm based on the EAET approximation to simulate non-Markovian quantum dynamics at a finite temperature. The algorithm is numerically stable and takes advantage of both quantum and classical computing. The wave function overlap evaluated in eq 12 can be handled with a linear number of qubits on a quantum computer, and the Monte Carlo sampling can be performed efficiently on a classical computer. The ensemble averaging effectively diminishes the impact of the shot noise. In addition, since each Ehrenfest trajectory originating from the Monte Carlo points can be propagated independently, the EAET algorithm can be implemented parallelly on the NISQ devices. Furthermore, the algorithm can be well adapted to treat anharmonic bath and nonlinear system-bath coupling and holds promise to extend the realm of simulation to multistate systems by employing the adaptive variational ansatz for shallow circuit construction.^36,37^

## Supporting Information Available

Transparent Peer Review report available (PDF)
