# Supplementary material for: Variational Quantum Algorithm for Non-Markovian Quantum Dynamics Using an Ensemble of Ehrenfest Trajectories
Source: J Phys Chem Lett. 2025 Jan 22;16(4):1001–6. doi: 10.1021/acs.jpclett.4c03431 (PMC11789130; doi:10.1021/acs.jpclett.4c03431)
Supplement: Supplementary file 1 — jz4c03431_si_001.pdf [file jz4c03431_si_001.pdf]

jz-2024-03431k.R1

Name: Peer Review Information for "Variational quantum algorithm for non-Markovian quantum dynamics"

First Round of Reviewer Comments

Reviewer: 1

Comments to the Author

Non-Markovian effects arising from system-environment coupling play a crucial role in understanding charge and exciton dynamics in the condensed phase. This study introduces a novel quantum algorithm for non-Markovian quantum dynamics, developed within the framework of the quantum-classical approximation and path integral representation, known as ensemble-averaged Ehrenfest trajectories (EAET). The performance of the algorithm is evaluated using a representative spin-boson model with two parameter sets. This research provides valuable insights for scholars in the field, encouraging further exploration, particularly in quantum simulations of non-Markovian dynamic effects.

Here are some questions and comments for the authors:

1. Based on the two sets of parameter tests conducted in this work, could the authors clarify the applicable range of the EAET-based quantum algorithm, particularly in relation to parameters such as bath temperature and the strength of the system-bath coupling?
2. In the spin-boson model, a single qubit suffices, enabling the use of an exact ansatz. To better showcase the advantages of this quantum algorithm, its performance—such as convergence, ansatz optimization, and circuit depth—should be investigated in multistate models like the exciton model, especially in complex systems such as the Fenna–Matthews–Olson (FMO) complex.
3. The manuscript provides a comprehensive discussion of the proposed methodology; however, it could be beneficial for the authors to briefly acknowledge alternative quantum algorithms for simulating non-Markovian quantum dynamics, such as the hierarchical equations of motion (Phys. Rev. A 110, 032620) or the stochastic Schrödinger equation (arXiv:2404.10655). Including these references, even optionally, would offer valuable context for readers and emphasize the distinctive aspects of the authors' approach.

Reviewer: 2

Comments to the Author

In this paper, Wang and coworkers present a new quantum algorithm for the simulation of non-markovian dynamics. The essence of their approach finds inspiration from Makri's earlier work on ensembles of classical trajectories, and uses ensemble averaging over erenfest trajectories to introduce non-markovian behavior. The implementation into a quantum setting, uses the p-VQD approach from Guissepo and coworkers which has several features which are attractive from an efficiency perspective. This work is interesting, and novel (as far as I can tell) and should be published after addressing the following minor concerns.

1. The EAET method can directly be extended to non-linearly coupled baths, and only requires the initial Wigner function to be available. As someone not too familiar with the details, how strict is this requirement? Perhaps adding some context here would help the readers understand how general the proposed method is.

2. I think the simulation results could be described more clearly to help readers. Personally, it took me a long time to understand what each line meant, and how I should compare the different simulation results. For instance, I was confused by the seemingly perfect agreement between the EAET (10k IC) and EAET(1 IC). It took me a while to realize that the black line (denoted by 1 IC) refered to the p-VQD and simulator results. Assuming I'm now understanding the plots correctly, I think the results are quite exciting, particularly that shot noise can be washed out by the initial condition sampling.

3. Several grammatical errors which should be taken care of

4. While the format of jpcl does encourage brevity, more details about the derivations would be helpful for the readers - a supporting information document would be ideal for this.

5. How should we expect the algorithm to scale? A single 2-level system seems capable of demonstrating your key ideas, but having a few more qubits would really improve the paper, particularly when it comes to the question "does the sampling error still cancel out during the ensemble averaging?"

Author's Response to Peer Review Comments:

The authors would like to thank the reviewers for useful comments. All the questions are appropriately addressed.The authors sincerely thank the reviewers for constructive comments. All the questions raised by the reviewers are properly addressed.

Response to Reviewer One:

1. We add a sentence to address the validity of the EAET algorithm: "The EAET approximation becomes more accurate as the temperature increases or the coupling strength becomes smaller, since in those regimes, the free propagation term in equation (5) dominates."
2. We briefly address this issue by adding a sentence to the end of the manuscript: "the algorithm ... holds the promise to extend the realm of simulation to multi-state systems by employing the adaptive variational ansatz for shallow circuit construction." The demonstration with a multi-site model such as the FMO deserves another major effort, including strategies to choose the best variational ansatz and the optimization on a higher dimensional parameter space. This research is currently undergoing in my group.
3. These two references are included as Reference 13 and 14.

Response to Reviewer Two:

1. A paragraph is added: "the framework can be equally adapted to non-linear coupling and anharmonic environment. The reason is that for an anharmonic trajectory, the equation of motion can be written in a analogous way as in equation (4), which is composed of a free propagation part and a back-reaction part. The free propagation can be easily solved numerically for any potential. The back-reaction part arises from the system-bath interaction and the analytical form is generally not known. However, we can well approximate the anharmonic back-reaction with the harmonic back-reaction (HBR) expressed in equation (4),[22,25] due to the fact that the back-reaction is largely a zero-point energy effect associated with the bottom of the potential well,[26] which in most cases can be very approximated to be harmonic. Therefore, the EAET approximation for anharmonic bath is no different from the expression of equation (5), except to replace the free propagation part with the numerical anharmonic one. The anharmonic bath Wigner distribution can be obtained by methods such as adiabatic switching.[27]"
2. Table 1 is added to succinctly summarize the results and methods. Also, a paragraph is added to emphasize the role of each curve: "To facilitate better reading the plots, we summarize the abovementioned methods and results in Table 1. The comparison between "Exact" and "Ehrenfest" serves the purpose to gauge the accuracy of the EAET approximation; the comparison between "Ehrenfest" and "p-VQD" aims to assess the accuracy of the circuit ansatz and the gradient descent algorithm; and the comparison between "p-VQD" and "Simulator" gives the impression about the measurement shot noise."
3. They have been corrected.
4. We have made a few changes in describing the equations so that the flow becomes easier to follow.
5. This is an important question to address; however, it deserves a separate endeavor. But the authors have made more explicitly at the end of the manuscript that the adaptive variational quantum algorithm is a good candidate for constructing multi-qubit circuits.

Name: Peer Review Information for "Variational quantum algorithm for non-Markovian quantum dynamics using ensemble of Ehrenfest trajectories"

Second Round of Reviewer Comments

Reviewer: 1

Comments to the Author

From the reply, it is clear that this algorithm works effectively for parameters within the semiclassical regime, such as those in Ehrenfest or surface hopping dynamics. Therefore, I suggest emphasizing this point in the title and conclusion to prevent misunderstanding the EAET algorithm as a general numerically exact method for non-Markovian quantum dynamics.

Author's Response to Peer Review Comments:

Based on the reviewer's recommendation, we have added "using ensemble of Ehrenfest trajectories" to the title as well as to the first sentence of the conclusion.
